# Seasonal synchronization and unpredictability in epidemic models with waning immunity and healthcare thresholds

**DOI:** 10.1038/s41598-025-01467-4

**Published:** 2025-05-17

**Authors:** Veronika Eclerová, Deeptajyoti Sen, Lenka Přibylová

**Affiliations:** 1 Department of Mathematics and Statistics, Faculty of Science, Masaryk University, Kotlářska 2, 611 37 Brno, Czech Republic; 2 Department of Informatics, Faculty of Business and Management, Brno University of Technology, Kolejní 2906/4, 612 00 Brno, Czech Republic

**Keywords:** SIRS model, Seasonality, Bifurcation, Chaos, Quasiperiodicity, Applied mathematics, Infectious diseases

## Abstract

This paper explores a model integrating healthcare capacity thresholds and seasonal effects to investigate the synchronization of epidemic cycles with seasonal transmission rates, using parameters reflective of the COVID-19 pandemic. Through bifurcation analysis in the epi-seasonal domain, we identify regions of significant seasonal synchronization related to transmission rate fluctuations, waning immunity, and healthcare capacity thresholds. The model highlights four sources of unpredictability: chaotic regimes, quasiperiodicity, proximity to SNIC or transcritical bifurcations, and bistability. Our findings reveal that chaotic regimes are more predictable than quasiperiodic regimes in epidemiological terms. Synchronizing outbreaks with seasonal cycles, even in chaotic regimes, predominantly results in significant winter outbreaks. Conversely, quasiperiodicity allows outbreaks to occur at any time of the year. Near eradication unpredictability aligns with historical pertussis data, underscoring the model’s relevance to real-world epidemics and vaccine schedules. Additionally, we identify a bistability region with potential for abrupt shifts in disease prevalence, triggered by superspreading events or migration.

## Introduction

In the field of mathematical epidemiology, compartmental models, such as the Susceptible-Infectious-Removed-Susceptible (SIRS) model, are employed to describe the dynamics of epidemic outbreaks caused by infectious diseases. Traditionally, the behavior of the SIRS model can be characterized by its endemic or trivial equilibria and their stability^1^. More recently, researchers have also explored endemic cyclic dynamics driven by various parameters. Previous studies have linked such cyclic dynamics to factors such as vaccination, waning immunity, limited treatment, and behavioral changes^2–6^, as well as seasonal effects^7,8^. In contrast to the analysis of equilibrium dynamics as parameters change, understanding cyclic dynamics is a more intricate and less explored phenomenon, although it is an important phenomenon that occurs, for example, in many re-emerging respiratory disease outbreaks. Typically, multiple factors contribute to the occurrence of cyclic dynamics in the epidemiological system under study, and these factors may interact to produce even more complex dynamics.

A fundamental concept explored in this paper is how the interaction between epidemic dynamics and seasonal parameters determines whether an epidemic becomes seasonal. Understanding disease seasonality remains insufficiently addressed^9^, and our results highlight that the interplay between epidemic progression and seasonal variations significantly influences the emergence of seasonality. Notably, neither COVID-19 nor the Spanish flu initially exhibited seasonal patterns^10,11^, yet COVID-19 now appears to be becoming seasonal, much like the H1N1 influenza A, which has long been established as seasonal^12^. The interaction between epidemic dynamics and seasonality has also been observed recently through the resurgence of influenza, which aligns with seasonal patterns after the lifting of COVID-19 non-pharmaceutical interventions^13^. Similarly, we show that phenomena near SNIC bifurcation resemble those observed during the global vaccination rollout against pertussis^14,15^.

To investigate these dynamics, we focus on modeling recurrent epidemic outbreaks, with periodicity influenced by three main factors: limited treatments, waning immunity, and the seasonal variation in disease spread. We employ a model^2^, which incorporates a healthcare capacity threshold. Our investigation of the model is based on the premise that there exists a threshold for limited treatment, arising from healthcare capacity or the availability of medical treatment. We also take into account the fact that some individuals may perceive the disease as less severe, leading them to forgo seeking healthcare. Nevertheless, we assume that the average duration of infectiousness is shorter with available medical treatment. However, as the infected population reaches a certain threshold, it begins to grow. Subsequently, it remains at a shifted, higher level. The authors of the original paper^2^ thoroughly studied the equilibria of the model depending on parameter values. However, their analysis of limit cycle dynamics is limited, leaving room for a more comprehensive investigation of oscillatory behavior, which we address in our study. Additionally, we include the seasonal effect in the transmission rate using a standard approach^7^. A plausible scenario is that climate change may alter the strength of these seasonal effects, potentially impacting the dynamics of disease spread^16^.

Within the realm of dynamical systems modeling, the emergence of a limit cycle dynamic is associated with the Hopf bifurcation. Concurrently, the manifestation of two distinct endemic cycles independently leads to the establishment of dynamic behavior on an invariant torus, commonly referred to as a secondary Hopf bifurcation, torus bifurcation or Neimark-Saker bifurcation. Typically, the dynamics observed on an invariant torus exhibit either quasi-periodic or periodic characteristics. Periodic dynamics are closely connected to the concept of phase locking, which can be comprehensively studied through the analysis of resonances and resonance tongues^17–19^.

In the present study, we conduct an in-depth analysis of the SIR model, taking into account healthcare capacity thresholds, waning immunity and seasonal transmission rates. This analysis is executed through the utilization of detection and continuation techniques for bifurcation analysis, employing the software tools Matlab^20^ and MatCont^21,22^.

The paper is organized as follows: The “Methods” section includes a general derivation and description of the model, along with settings for model parameters related to respiratory disease epidemics, using covid-like diseases as a case study, and analysis techniques based on bifurcation theory. The “Results” section presents bifurcation analyses of the introduced systems, both without and with seasonality, explaining the emergence of seasonally synchronized endemic cycles. This analysis also elucidates various observed epidemic phenomena, such as eradication (as observed in historical pertussis data) that started from full one-year period synchrony, followed by multiannual cycles to eradication, or the potential for abrupt large outbreaks that may occur in regions of bistability, as well as the chaotic dynamics. The simulations in the last two sections incorporate stochastic dynamics, introducing four different mechanisms of unpredictability that arise from the presented analysis. In the “Discussion” section, we summarize the study, outline some advantages and limitations of our approach, and compare it with real-world epidemic phenomena.

## Methods

### Limited treatment model

We model the dynamics of recurrent epidemics due to limited treatment by a system of differential equations, which represents a compartmental SIRS (Susceptible-Infectious-Removed-Susceptible) model with a nonlinear recovery rate. We omit the exposed compartment of infected and non-infectious individuals due to the simplicity of the model, since adding this compartment has no effect on the non-linear mechanisms we study. The following parameters can be defined:

**Parameters:**

- $\beta$: The transmission rate, specifying the probability of disease transmission per time from infectious to susceptible individuals which includes the average number of contacts per person per time and the probability of transmission.
- $\gamma$: The recovery rate, indicating the fraction of infected individuals recovering without treatment per unit of time.
- $f(K, \eta , r, I)$: The function describing acceleration of removed rate. The function is defined by following variables and parameters: *K* - a population threshold (healthcare availability), $\eta$ - a maximal acceleration rate, *r* - a steepness by which the threshold *K* is reached, and *I* - the current infectious proportion. For more details see “Acceleration of removed rate” section.
- $\nu$: The waning immunity rate, representing the rate at which recovered individuals lose immunity or exit the removed compartment.

**Equations:** The system of differential equations is as follows:

1 $$\begin{aligned} \begin{aligned} \frac{dI}{dt}&= \beta S I - \gamma f(K,\eta ,r,I) I, \\ \frac{dR}{dt}&= \gamma f(K,\eta ,r,I) I - \nu R, \\ S&= 1 - I - R. \end{aligned} \end{aligned}$$

The selection of system parameters in our study is based on a conceptual approach rather than direct experimental data. However, we made an effort to align this conceptual principle with realistic parameter values to enhance the understanding for researchers who work with epidemiological data. Our predictive model^23^, which was also part of the European Centre for Disease Prevention and Control (ECDC) ensemble^24^ with high predictive accuracy, demonstrated our experience with real data, where we thoroughly discussed the choice of the SIR(S) model and the methodology for parameter estimation. Consequently, the analysis presented in this paper is conducted within a parameter range that corresponds to a realistic epidemiological scenario.

Parameters are chosen to evoke the COVID-19 disease^23^. The transmission rate, $\beta$, is set to small hundreds, which corresponds to the serial interval in days^25^. It is heavily influenced by the contact rate; however, we assume an average long-term contact rate within the community, excluding short-term periods of non-pharmaceutical interventions, such as lockdowns and mandatory masking. Since both the contact rate and the serial intervals are stochastic, we will also demonstrate how this stochasticity interacts with seasonality. Specifically, we will explain four different mechanisms of unpredictability in “Unpredictability and stochastic differential equations” section. Health-seeking behavior is strongly associated with the severity of illness^26^, while the severity of COVID-19 significantly depends on age and comorbidities. Therefore, we assume that the percentage of people seeking healthcare or isolating themselves is small, specifically, *K* is in small units of percentages. The recovery rate, $\gamma = 36$, indicates an approximately 10-day infectious period^27,28^. The rate of waning immunity $\nu$ can be considered in small units or could be even higher for post-delta SARS-CoV-2 variants, which have a high percentage of reinfections and evidence of post-vaccination and post-infection protection decline^29^. For simplicity, we omit mortality by the disease assuming this compartment would be very small with respect to the susceptible compartment.

### Acceleration of removed rate

After infection with an infectious disease, individuals may experience a spectrum of symptoms, ranging from mild to severe (for COVID-19, see meta-analyses^30,31^, clinical studies^32,33^ or nation-wide data analyses^29,34^). This variation is evident in the choices individuals make regarding seeking healthcare or entering isolation, with those experiencing severe symptoms more frequently opting to seek medical attention^26^. If they take this course of action, they can either recover or, at the very least, be isolated, preventing further spread of the disease. It is worth noting that the proportion of the population at risk of developing severe illness is probably relatively small^26^.

From a mathematical perspective, a higher removal rate $\gamma$ is associated with the proportion of people seeking healthcare or isolating. We follow a model^2^, where acceleration was defined using a piece-wise constant function. We generalize this approach by using its standard continuous approximation. The function

2 $$\begin{aligned} f(K, \eta , r, I) = \frac{\eta + \eta e^{-r (I-K)}}{\eta + e^{-r (I-K)}} \end{aligned}$$

governing the acceleration of the removal rate $\gamma$ is determined by the following variables and parameters: *K*, signifying the proportion of individuals seeking healthcare or isolation; $\eta$, representing the maximum acceleration rate; *r*, indicating the steepness with which the threshold *K* is attained; and *I*, denoting the current infectious proportion.

**Fig. 1:**
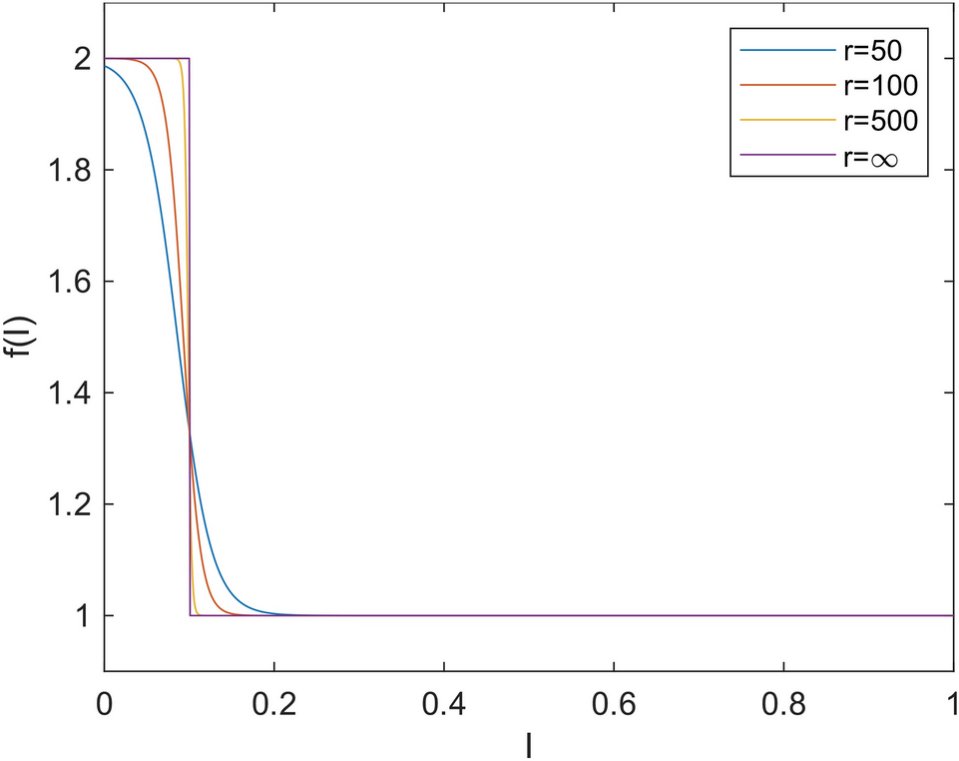
Acceleration of removed rate $f(K, \eta , r, I)$ for $K=0.1$, $\eta =2$ and various values of *r*. The step-wise function *f* from the original paper^2^ is smoothed on interval around (0.047, 0.140) reaching there values 1.99 and 1.01.

Fig. 1 illustrates the function *f* across various values of the parameter *r*. It is evident that, for higher values of *r*, the function tends towards a step function. This implies the existence of two potential removal rates $\gamma$. From that perspective, the function *f* is considered as an approximation of the step function. When analyzing the model using continuation software like MatCont^21,22^, it is crucial for the function to be continuous. Also in reality, the function is likely not a step function at some given threshold *K*; rather, the threshold *K* may exhibit some variation, and a continuous function is a better fit for such variations. For the purpose of this paper we use $r=100$.

### Limited treatment and seasonal transmission rate model

Following a previous research^7^, the transmission rate $\beta$ may exhibit variation over the course of a year, influenced by seasonal changes. This variation can be attributed to the seasons, where during the summer, the transmission rate tends to be lower due to increased room ventilation, reduced indoor occupancy, enhanced outdoor activities, and decreased contact rate resulting from summer holidays, etc. In contrast, during winter, individuals tend to stay indoors, and the colder weather makes them more susceptible to the disease, leading to an increase in the transmission rate.

Therefore $\beta (t)$ is time-dependent periodic function:

3 $$\begin{aligned} \beta (t)=\beta _0(1+\alpha \cos (2\pi T t)), \end{aligned}$$

where $\alpha \ge 0$ is the amplitude of the seasonal oscillation as percentage of $\beta _0$, $T=1$ is the period of function $\beta (t)$, and *t* is time with year-long scaling. Using time-dependent transmission rate, described by Eq. (3), we obtain system of following differential equations

4 $$\begin{aligned} \begin{aligned} \frac{dI}{dt}&= \beta _0 \left( 1 + \alpha \cos (2\pi T t)\right) S I - \gamma f(K,\eta ,r,I) I, \\ \frac{dR}{dt}&= \gamma f(K,\eta ,r,I) I - \nu R, \\ S&= 1 - I - R. \end{aligned} \end{aligned}$$

### Embedding

We model the effect of seasonality with a harmonic oscillation driving the epidemic sub-system, which naturally displays a limit cycle (as seen in Eq. (4) and reference^2^), resulting in a non-autonomous system. We can utilize a previously described embedding method^35–37^ for harmonically driven oscillators with ease to a generalized higher-dimensional autonomous system. Suppose the system can be expressed in the following form:

5 $$\begin{aligned} \dot{\textbf{x}} = \textbf{f}(\textbf{x}, \alpha \cos (2\pi T t), \pmb {\beta }), \end{aligned}$$

where $\textbf{f}:\mathbb {R}^{m+p+1} \rightarrow \mathbb {R}^m$ is a function smooth enough, and $\pmb {\beta } \in \mathbb {R}^p$ are given parameters. $\alpha \cos (2\pi T t)$ denotes a harmonic external force with amplitude $\alpha$ and frequency *T*. The system (5) can be embedded into an autonomous system:

6 $$\begin{aligned} \begin{aligned} \dot{\textbf{x}}&= \textbf{f}(\textbf{x}, u, \pmb {\beta }),\\ \dot{u}&= 2\pi T(A u - v - u(u^2 + v^2)),\\ \dot{v}&= 2\pi T(u + A v - v(u^2 + v^2)), \end{aligned} \end{aligned}$$

The two-dimensional driving sub-system incorporated for $(u,v)^T$ is in the normal form of a super-critical Hopf bifurcation. When *A* is negative, it possesses a stable equilibrium at the origin, while for positive *A*, a stable limit cycle of the form $S = \{(u,v):u^2+v^2=A\}$ emerges. Consequently, when $A = \alpha ^2$, the dynamics of the system (6) on the invariant manifold $\mathbb {R}^m\times S$ become topologically equivalent to the system (5) dynamics. The synchronization of a driven oscillator (the epidemic endemic cycle) with driving oscillator (the seasonal cycle) in the multidimensional state space corresponds to the emergence of a unique limit cycle, while the non-synchronous oscillations of the two oscillators create a quasi-periodic trajectory on the torus or more complex dynamics. The asymptotic stability of cycle *S* (driving oscillator) ensures favorable numerical properties for the continuation of bifurcation manifolds^35–37^, particularly in software packages like MatCont^21,22^. Additionally, the autonomous nature of the system enables the computation of the Lyapunov spectrum, which will be discussed in the following “Analysis techniques” section.

In this case, we can embed model (4) into a new generalized system

7 $$\begin{aligned} \begin{aligned} \frac{dI}{dt}&= \beta _0 (1 + u) S I - \gamma f(K,\eta ,r,I) I, \\ \frac{dR}{dt}&= \gamma f(K,\eta ,r,I) I - \nu R, \\ \frac{du}{dt}&= 2\pi T(A u - v - u(u^2 + v^2)), \\ \frac{dv}{dt}&= 2\pi T(u + A v - v(u^2 + v^2)), \\ S&= 1 - I - R, \\ A&= \alpha ^2 \text { for } A \ge 0. \end{aligned} \end{aligned}$$

### Analysis techniques

We provide bifurcation analysis of system (1) in MatCont^21,22^, version 7p4. Bifurcation refers to a qualitative change in the dynamics of the studied system as a parameter or set of parameters varies. These changes often involve, for example, the emergence, destruction, or alteration of equilibrium points, periodic orbits, or other invariant sets^18,19^. In this paper, we primarily focus on the emergence of cycles. We interpret the limit cycle dynamics as a periodic epidemic outbreaks^1^.

It is crucial to note that bifurcation analysis, although computed numerically, fundamentally differs from simulation. Instead of observing the system’s behavior for specific parameter values, we use a continuation approach to systematically trace the boundaries where qualitative changes occur. This continuation process involves formulating the detection of bifurcation points as a system of algebraic equations, which are then solved using a Newton-like predictor-corrector method. This methodology allows us to accurately and efficiently map out bifurcation boundaries within the parameter space.

In epidemiology, researchers often analyze the basic reproduction number, which indicates whether a disease has the potential to spread. If this number exceeds one, the disease can spread. From the perspective of bifurcation theory, this phenomenon can be elucidated through the transcritical bifurcation. Transcritical bifurcation involves a switch in stability between two equilibrium states. In this scenario, it occurs between a trivial equilibrium (representing a disease-free state) and an endemic equilibrium. Transcritical bifurcation is not the only mechanism how the basic reproduction number exceeds one (see^1^), but it is the only relevant one for our model (1).

As our primary aim is to study the emergence of cycles, we focus on one-parameter bifurcations starting with the Hopf bifurcation. In its super-critical version, it allows a stable limit cycle to arise from a stable equilibrium, that changes stability on the Hopf manifold. The sub-critical version has exactly opposite stability, it gives birth of an unstable limit cycle from unstable equilibrium. Along the Hopf manifold, we identified generalized Hopf points (GH). At these generalized points, the stability of the studied limit cycle changes, and a region of bistability between the stable limit cycle and the endemic equilibrium arises ^19^.

A second mechanism of a limit cycle emergence is the limit point of cycles bifurcation when a pair of limit cycles, one stable and one unstable, collide and disappear. The existence of this bifurcation is guaranteed at least in a neighborhood of the two parameter GH bifurcation^19^.

A third mechanism is the saddle-node infinite cycle (SNIC) bifurcation, which is characterized by limit cycle, which period becomes longer and longer, finally it splits on a homoclinic orbit. This bifurcation is guaranteed at least in a neighborhood of the two-parametric Bogdanov-Takens bifurcation^19^.

By introducing seasonal transmission rate model (4) the dynamics become more complex. We study an interaction of two periodic forces in the model (4), one given by seasonal transmission rate and the second by the existence of limit cycle dynamics in model (1). We identified there important dynamical scenarios:

1. The model may undergo a cascade of period-doubling and end in chaotic regime.
2. The model may exhibit dynamics on an invariant torus. Seasonally synchronized cycles in case of strong enough seasonality in transmission rate/quasiperiodicity if not.
3. Due to bistability in model (1), we may encounter bistability also in model (4).

To investigate the phenomena described above, we employ the Poincaré sections^38^ and the Lyapunov exponent spectrum^39,40^, in addition to bifurcation analysis of system (7) in MatCont^21,22^, version 7p4.

A Poincaré section provides a method to convert a continuous dynamical system into a discrete one. This process involves selecting a lower-dimensional surface, known as the Poincaré section, which intersects the trajectory of the continuous system. Equivalently, the selection of this section can be guided by criteria such as maximum and minimum values of certain variables along the trajectory, or a section at given times of the period. The points where the trajectory intersects this section are used to define a discrete sequence of states for the system.

Lyapunov exponents are essential mathematical measures that quantify the divergence or convergence of trajectories in dynamical systems, reflecting the system’s sensitivity to initial conditions. When two points are initially proximate in phase space, a positive Lyapunov exponent denotes exponential divergence in their separation, indicating chaotic dynamics, whereas a negative exponent implies convergence and stability. Understanding the mechanisms underlying the emergence and disappearance of chaotic dynamics in infectious disease outbreaks is crucial. Early investigations from the initial stages of the COVID-19 pandemic have pointed to the potential presence of such behavior^41^. In one-dimensional systems; there is generally a single Lyapunov exponent; however, higher-dimensional systems possess a Lyapunov spectrum, which comprises a collection of exponents indicative of stability over all directions. Lyapunov exponents offer valuable insights into a system’s stability and qualitative behavior, particularly near bifurcations that may lead to complex dynamics such as chaos; shifts in their sign or magnitude can signal critical transitions even when standard continuation methods are not feasible. To illustrate this, the following table 1 summarizes typical dynamical attractors along with their characteristic Lyapunov exponents and associated minimal embedding dimensions.

**Table 1:** Common types of dynamical attractors with their characteristic Lyapunov exponents and the minimum phase space dimension required for their occurrence.

Dynamical attractor	Lyapunov exponents	Min. dimension
Limit cycle	One zero, other negative	2
2D-Torus	Two zeros, other negative	3
3D-Torus	Three zeros, other negative	4
Chaotic attractor	One positive, at least one zero and other negative	3
Hyperchaotic attractor	At least two positive, at least one zero and other negative	4

To quantify the Lyapunov spectrum^39^ for a nonlinear system, one generally starts by numerically simulating the system’s dynamics while concurrently monitoring the evolution of minor perturbations to the system’s state across time. This entails the integration of the original nonlinear system alongside its corresponding variational equations, which describe the linearised dynamics of perturbations around a trajectory. The rate of progression of these perturbations is determined by the system’s Jacobian matrix, computed along the trajectory at each time interval. To ensure numerical stability and avoid vector collapse, the Gram-Schmidt orthonormalization method (or QR decomposition) is often applied to the evolving perturbation vectors. With time, the average exponential growth or decay rates of these orthonormalised vectors converge to the Lyapunov exponents, resulting in the whole spectrum. We employ this technique to compute the spectrum by Govorukhin^40,42^.

In the last section, we introduce stochasticity into the transmission rate in the model (7). We study the following set of stochastic differential equations:

8 $$\begin{aligned} \begin{aligned} \text {d}I&= \beta _0 (1 + u) S I \text {d}t - \gamma f(K,\eta ,r,I) I \text {d}t + \sigma \beta _0 S I \text {d}W\\ \text {d}R&= \gamma f(K,\eta ,r,I) I \text {d}t - \nu R \text {d}t, \\ \text {d}u&= 2\pi T(A u - v - u(u^2 + v^2)) \text {d}t, \\ \text {d}v&= 2\pi T(u + A v - v(u^2 + v^2)) \text {d}t, \\ S&= 1 - I - R, \\ A&= \alpha ^2 \text { for } A \ge 0, \end{aligned} \end{aligned}$$

where *W* denotes Wiener process and $\sigma$ measures transmission rate non-seasonal stochastic variability, and simulate them using Milstein method^43^.

## Results

### Bifurcation analysis of the limited treatment model without seasonality

The emergence of a limit cycle in model (1) without seasonality is highly dependent on the threshold values of the acceleration of the removed rate (*K*), waning immunity ($\nu$), and transmission rate ($\beta$). Therefore, in this section, we conduct a bifurcation analysis of model (1) with respect to these parameters.

**Fig. 2:**
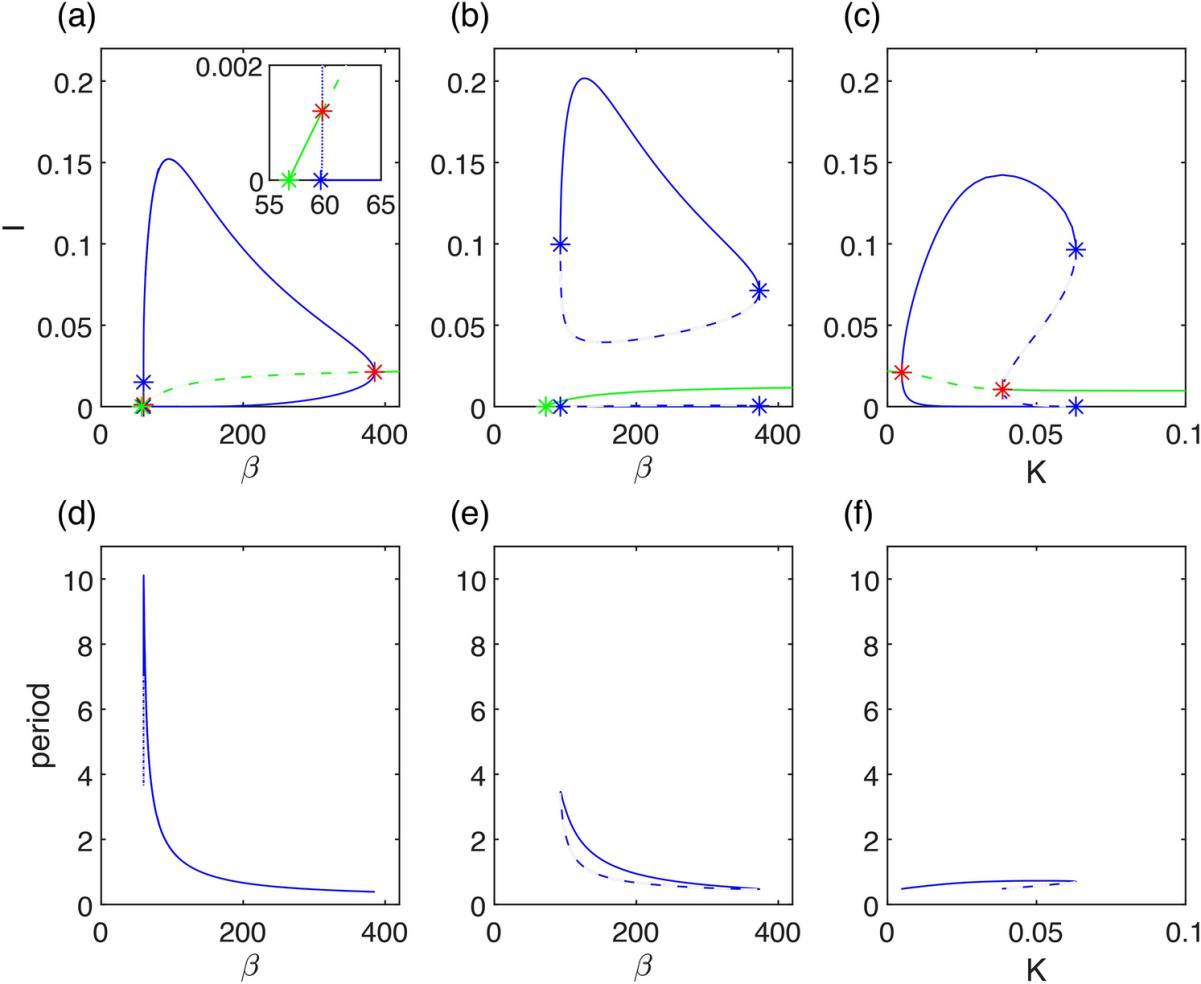
One-parameter bifurcation diagrams and corresponding periods: Curves indicate invariant sets: blue color—limit cycle, green color—equilibrium, full line—stable, dashed line—unstable. Stars indicate one-parameter bifurcations: blue—limit point of cycles, red—Hopf bifurcation, green—transcritical bifurcation. Parameters are set to $\gamma =36$, $\eta =2$, $r=100$, $\nu =1$, and for (**a)**, (**d**) $K=0.01$ with $\beta$ as the bifurcation parameter, (**b)**, (**e**) $K=0.05$ with $\beta$ as the bifurcation parameter, (**c)**, (**f**) $\beta =250$ with *K* as the bifurcation parameter.

**Fig. 3:**
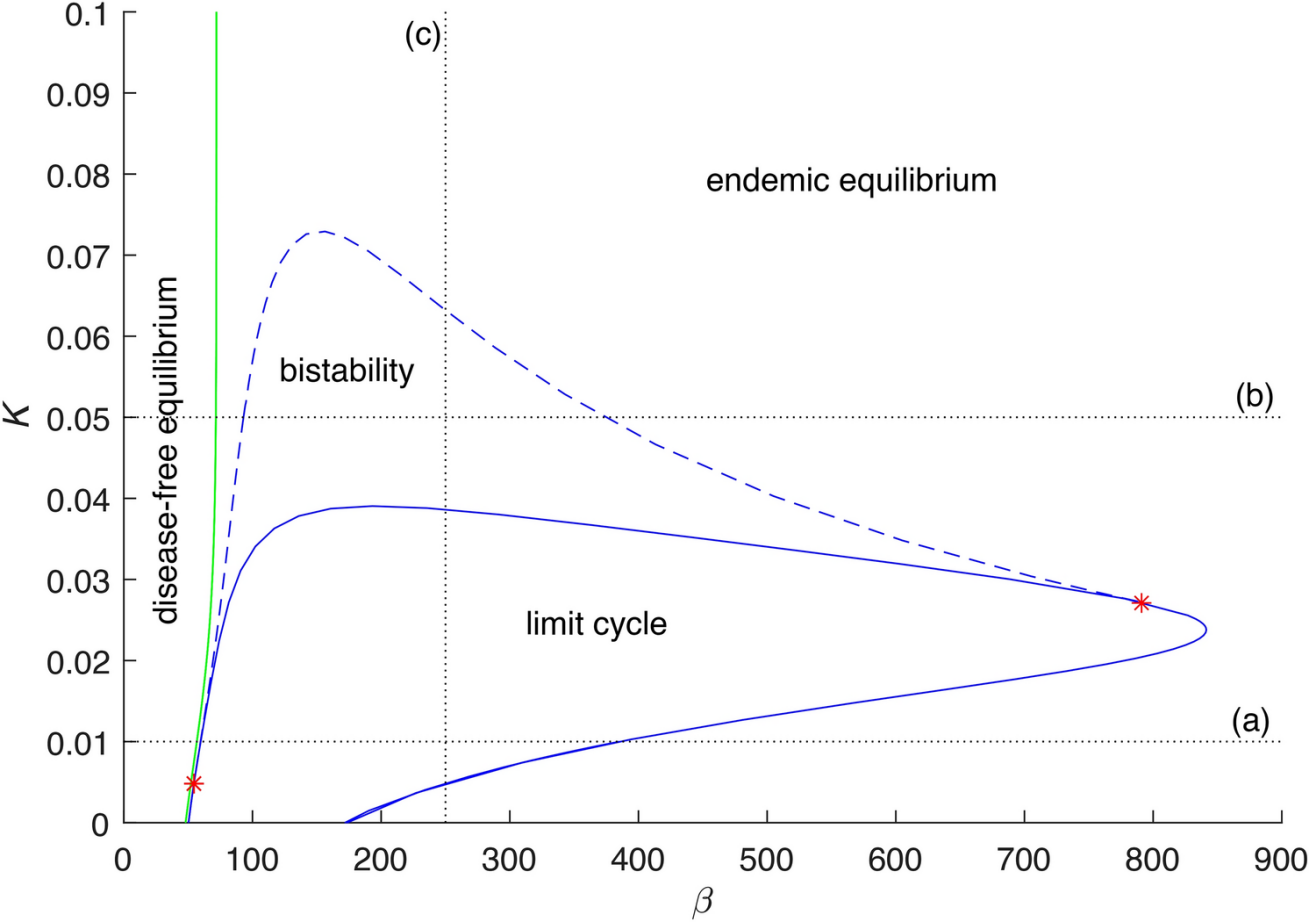
Two-parameter bifurcation diagram with $\beta$ and *K* as the bifurcation parameters, other parameters are $\gamma =36$, $\eta =2$, $r=100$, $\nu =1$. Dotted lines are referring to bifurcation diagrams in Fig. 2(a)–(c). Other curves denote one-parameter bifurcation manifolds: full blue line—Hopf, dashed blue line—limit point of cycles, full green line—transcritical bifurcation. Red stars denote generalized Hopf bifurcation points.

Initially, we discuss one-parameter bifurcation diagrams in Fig. 2(a), (b), with $\beta$ as the bifurcation parameter, considering different levels of *K*. For small values of $\beta$ the system has only one stable disease-free equilibrium. The system undergoes transcritical bifurcation which is a critical point related to the basic reproduction number $R_0=1$. As we cross this value, one endemic equilibrium emerges. For larger values of $\beta$ the model demonstrates a dynamic with a limit cycle, which arises through either a sub-critical Hopf bifurcation, or a super-critical Hopf bifurcation. Therefore, the limit cycle is either the only attractor or there is bistability between the limit cycle and the endemic equilibrium. The region of bistability is wider as *K* gets larger and then finally for large *K* the limit cycle dynamics does not emerge, see Fig. 3. In Fig. 2(c), you can see another plausible section of Fig. 3 that reveals how the bistability with respect to *K* emerges. It is a one-parameter bifurcation diagram with *K* as the bifurcation parameter. For small values of *K*, an endemic equilibrium exists, followed by the system undergoing a super-critical Hopf bifurcation and the system exhibiting limit cycle dynamics. For higher values of *K* the system undergoes sub-critical Hopf bifurcation and enters region of bistability, both the endemic equilibrium and the limit cycle are stable. The region of bistability ends on the limit point of cycles and the endemic equilibrium remains the only stable attractor.

The region of endemic cycle existence for $\beta$ varies considerably depending on the values of *K*, see Fig. 3. The other parameter that affects the existence of a limit cycle and its dynamics is $\nu$. Generally, the interval is broader for smaller values of $\nu$, but its topology remains similar for $\nu <3$, that is for waning longer than 4 months. The system exhibits Generalized-Hopf (GH) bifurcation for $\beta \approx 215.58$, $\nu \approx 1.35$. For $\nu$ smaller than the critical value for GH the system does not exhibit bistability, for larger values of $\nu$ it does (we assume $\beta$ smaller than 500). To interpret it with respect to the duration of post-infectious immunity, the endemic equilibrium can coexist with an endemic cycle only for waning faster then 3/4 of a year in case of this covid-like parameter setting. The system also exhibits the Bogdanov-Takens (BT) bifurcation point for $\beta \approx 57.00$, $\nu \approx 3.36$, two-parameter bifurcation that is related to another non-local bifurcation called the saddle-node infinite cycle (SNIC) bifurcation. It is crucial to note that the proximity to the SNIC bifurcation results in a significant change in the length of the period of the studied limit cycle within the examined interval, see Fig. 2(d)–(f). This factor plays a crucial role in the seasonal synchronization, as investigated in the subsequent “Bifurcation analysis of the limited treatment and seasonal transmission rate model” section. Again, we can interpret this with respect to the Acute Respiratory Infections (ARI). Getting close to this SNIC bifurcation value, the outbreaks become rare. For a seasonal disease with annual cycles, multiennial cycles may appear and at last the disease can seem to be almost eradicated for a long time. This scenario of gradual eradication is consistent with pertussis data^14,15^. In our analysis, we have concentrated on the long-term dynamics of model (1), which necessitates the inclusion of births and deaths. Hence, in Appendix A, we examine model (9) with no significant effect on the studied phenomena.

### Bifurcation analysis of the limited treatment and seasonal transmission rate model

In model (7), two periodic forces are competing. One arises from the limit cycle dynamics in model (1), and the second is associated with the seasonality of the transmission rate $\beta$. As discussed in the preceding section, the frequency of disease outbreaks, determined by the length of the limit cycle period, exhibits significant variability. Consequently, it can synchronize with the seasonal transmission rate.

**Fig. 4:**
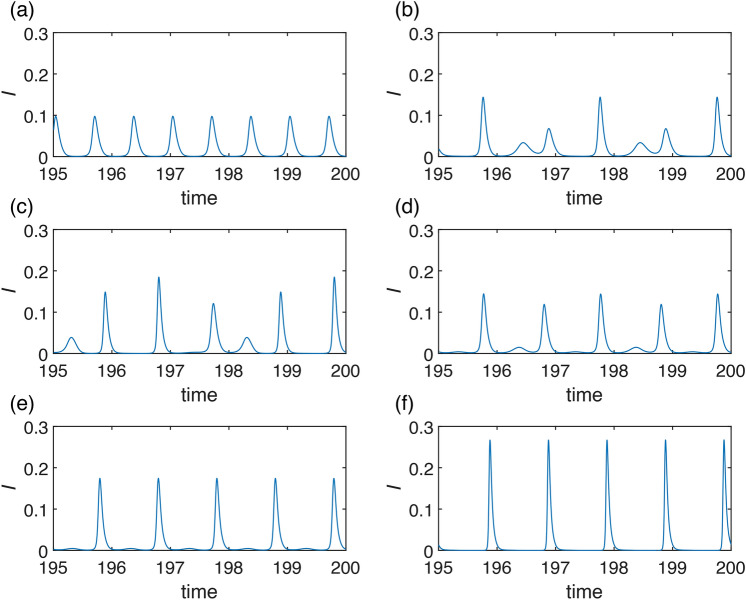
Attractors of models (1), (7) for parameters $\beta =200$ (resp. $\beta _0=200$), $K=0.01$, $\gamma =36$, $\eta =2$, $r=100$, $\nu =1$ and (**a**) with constant transmission rate, (**b)**–(**f**) with seasonal transmission rate with (**b**) $\alpha =0.3$, (**c**) $\alpha =0.35$, (**d**) $\alpha =0.39$, (**e**) $\alpha =0.45$, (**f**) $\alpha =0.6$ with initial conditions $I(0)=0.15$, $R(0)=0.65$.

**Fig. 5:**
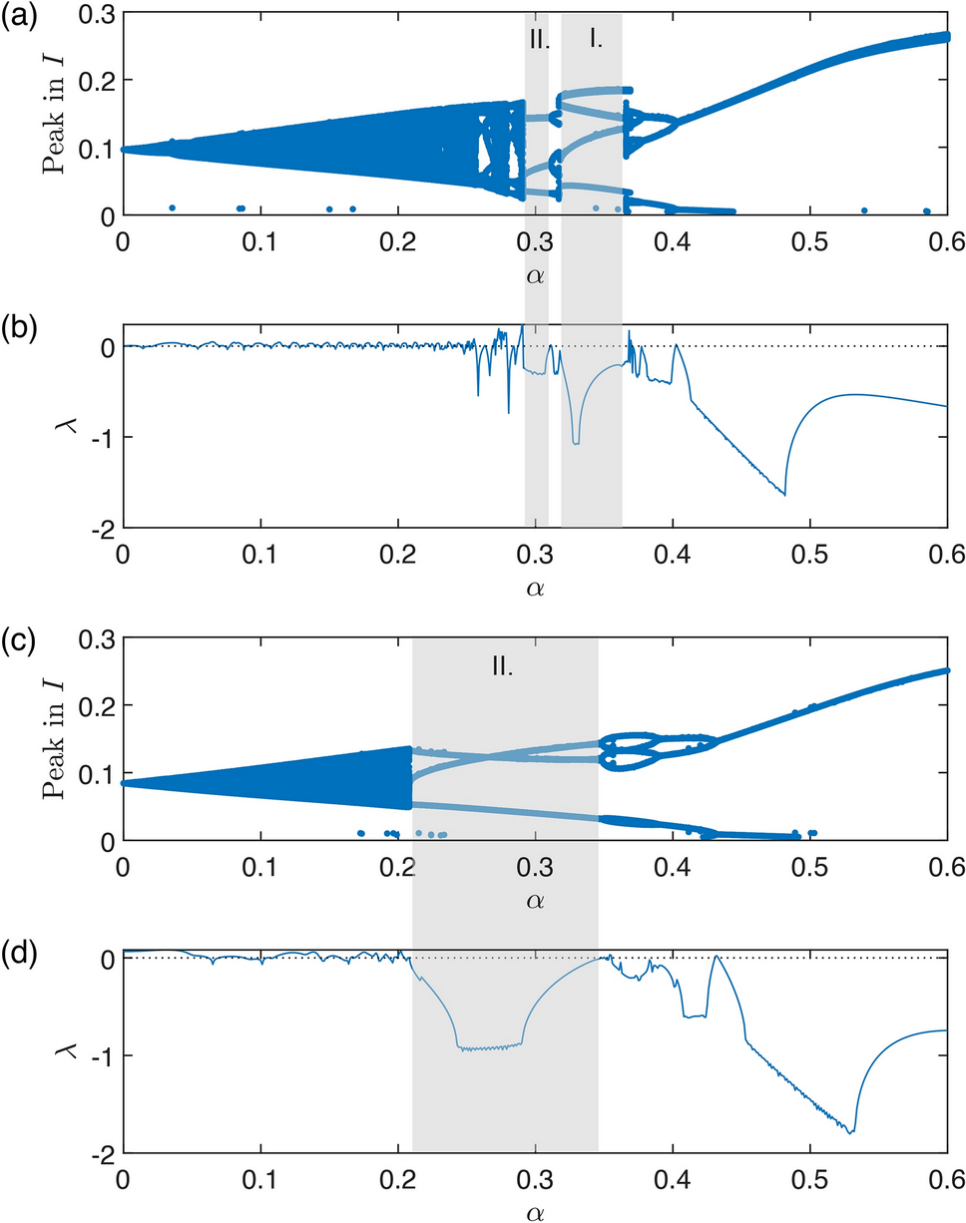
Bifurcation diagram of system (7) with respect to $\alpha$ as a bifurcation parameter and other parameters set as $\beta _0=200$, $\gamma =36$, $\eta =2$, $r=100$, $\nu =1$, $K=0.01$ in (**a**), (**b**) and $K=0.008$ in (**c**), (**d**) with initial conditions $I(0)=0.15$, $R(0)=0.65$. Estimated epidemic peak height (local maxima of *I*(*t*)) for each $\alpha$ are described in (**a**) and (**c**), by excluding transient 100 and with total time 200. (**b)**, (**c**) Lyapunov exponents: Since the initial conditions are set so to start on a stable limit cycle, we are excluding the one Lyapunov exponent that is always zero and one that is always negative. The Figure displays the maximal Lyapunov exponent out of the remaining two. In (**a**) and (**b**), the shaded regions mark the two largest periodic windows of the chaotic region. (**c)**, (**d**) While changing *K* only one periodic window stays.

Firstly, we show the synchronization using parameters $\beta _0=200$ (resp. $\beta =200$), $K=0.01$, $\gamma =36$, $\eta =2$, $r=100$, $\nu =1$. The natural frequency of the periodic orbit in the model (1) without seasonal transmission rate is approximately 0.66 year, see Fig. 4(a). For a significant amplitude of seasonal oscillations $\alpha$ in transmission rate, the system dynamics synchronize with the seasons and display a single winter peak, indicating 1:1 synchronization, See Fig. 4(f). As the amplitude $\alpha$ decreases, the system shows two epidemic peaks, with a prominent winter peak and a smaller summer peak, while still maintaining 1:1 synchronization, see Fig. 4(e). When $\alpha$ is set to approximately 0.4, the system undergoes a period-doubling bifurcation, resulting in two distinct types of dynamics in consecutive years. In one year, there are two epidemic peaks (a noticeable autumn peak and a smaller spring peak), while in the other year, there is either no spring peak or it is significantly smaller, See Fig. 4(d). With an even smaller amplitude of seasonal oscillations, the system undergoes a cascade of period doubling, eventually leading to a chaotic regime. Using bifurcation analysis, made possible by our approach involving an embedded torus in a multidimensional state space, we can identify this region with respect to parameters and explain the observed behavior, as detailed in the next paragraph. In Fig. 5, two distinct periodic windows are evident (marked by gray rectangles). The first window exhibits four epidemic peaks occurring across three consecutive years (Fig. 4(c)), while the second window displays three epidemic peaks occurring across two consecutive years (Fig. 4(b)). If $\alpha$ is less than 0.23, the system dynamics become quasi-periodic, lacking significant periodic windows, and consequently, it does not display synchronization, see Fig. 5(a), (b) and Appendix B. Since for values of parameter $\alpha$ around approximately 0.3 the system exhibits chaos, a small shift of parameter *K* from $K=0.01$ to $K=0.008$ causes a dramatic change in dynamics. Unlike in Fig. 5(a), (b), in Fig. 5(c), (d) you can see now only one periodic window displaying three peaks in two consecutive years.

**Fig. 6:**
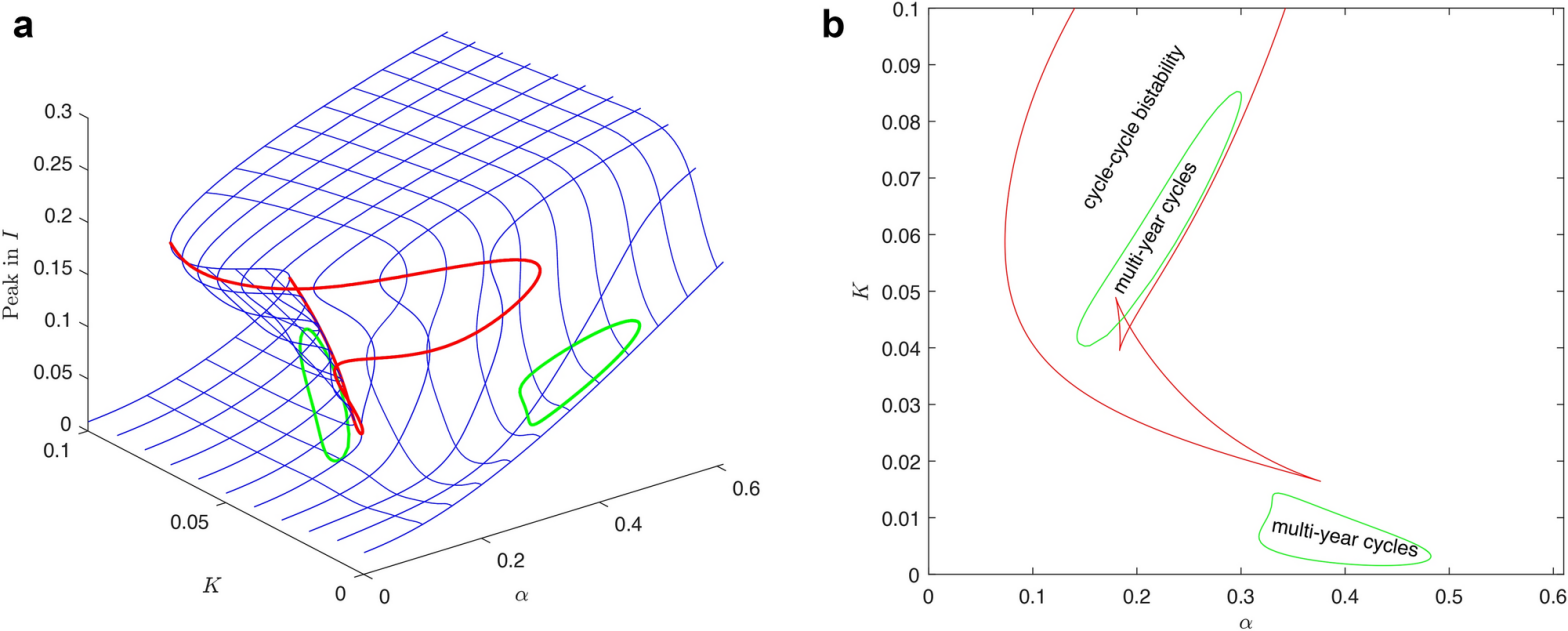
Bifurcation diagram of system (7) with $\alpha$ and *K* as parameter and other parameters are $\beta _0=200$, $\gamma =36$, $\eta =2$, $r=100$, $\nu =1$. The diagram shows bistability and multiyear-cycle regions. One-parameter limit-cycle continuations with respect to both $\alpha$ and *K* (blue), two-parameter LPC continuation (red), two-parameter PD continuation (green). (**a**) Depicts two-parameter bifurcations together with a two-dimensional limit-cycle manifold showing the epidemic peaks. (**b**) Shows a standard two-parameter bifurcation diagram that is a projection of (**a**) onto $\alpha \times K$ plane.

**Fig. 7:**
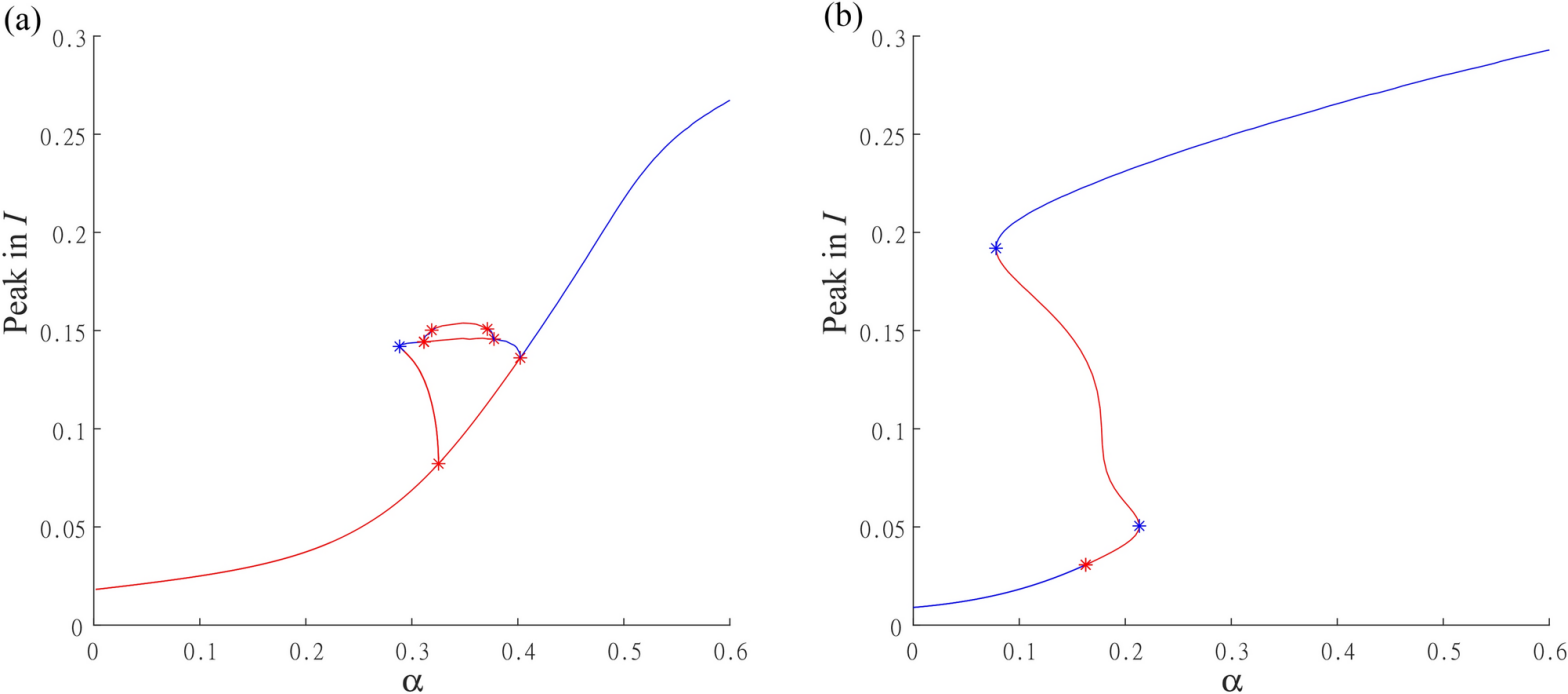
Two detailed sections of bifurcation diagram from Fig. 6 with (**a**) $K=0.01$ and (**b**) $K=0.05$ and other parameters are $\beta _0=200$, $\gamma =36$, $\eta =2$, $r=100$, $\nu =1$. Figures show continuations of cycles with respect to parameter $\alpha$ with stable cycles (blue line), unstable cycles (red line), LPC bifurcations (blue stars), and PD bifurcations (red stars). Figure (**a**) depicts a typical period doubling route to chaos near $\alpha = 0.4$ with 2-year and 4-year cycle branches continued from PD points. (**b**) Depicts typical bistability due to double fold of the cycle manifold.

**Fig. 8:**
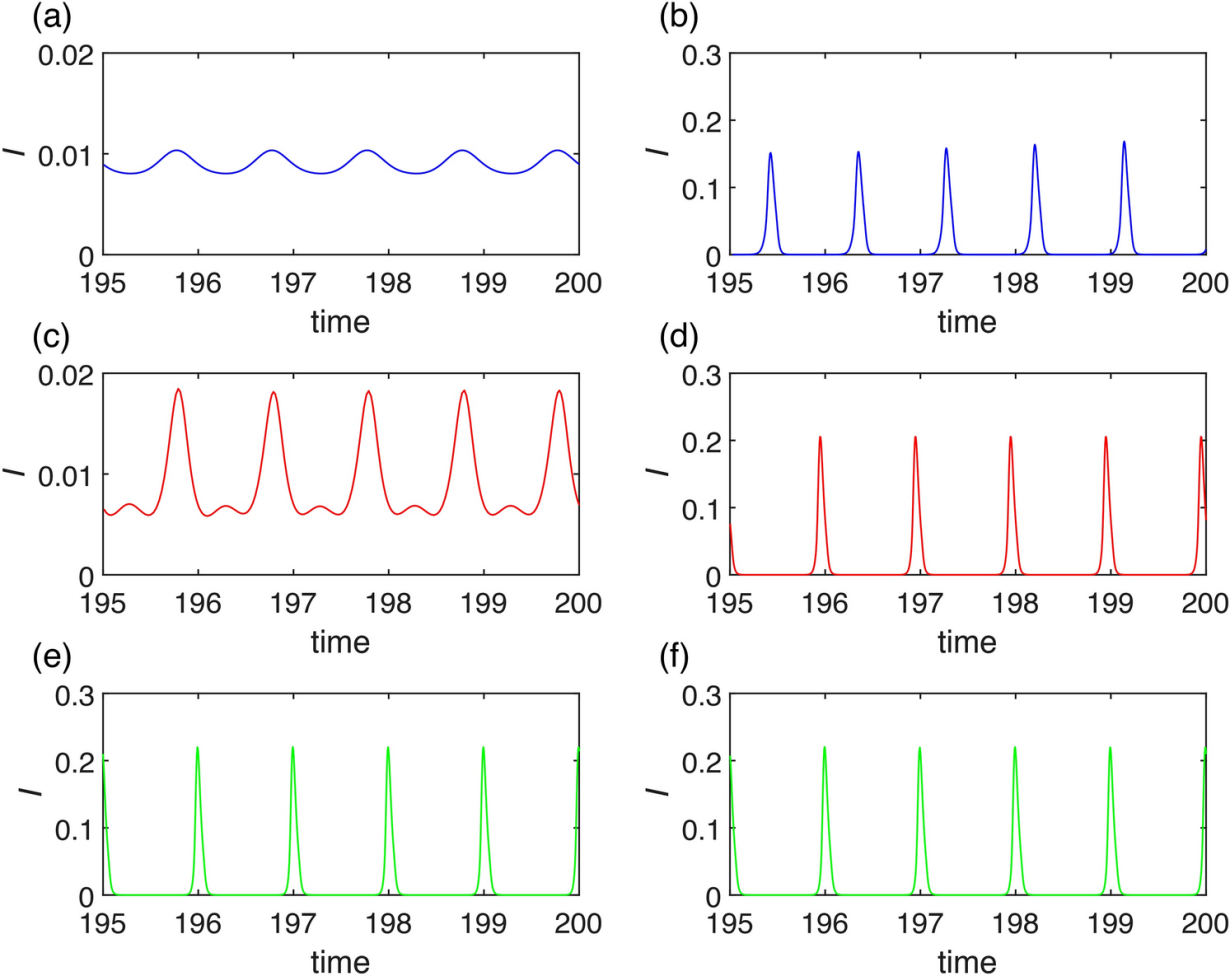
Bistability in model (7) for parameters $\beta _0=200$, $K=0.05$, $\gamma =36$, $\eta =2$, $r=100$, $\nu =1$ with seasonal transmission rates: $\alpha =0.025$ (blue) for (**a)**, (**b**) representing the case of seasonally fluctuating endemic equilibrium and quasiperiodicity on a torus (outbreaks are not synchronized with the season); $\alpha =0.1$ (red) for (**c)**, (**d**) representing the case of seasonally fluctuating endemic equilibrium and phase-locked cycle on a torus (outbreaks are synchronized with the season); and $\alpha =0.15$ (green) for (**d)**, (**e**) representing the case of a unique attractor – phase-locked cycle on a torus (outbreaks are synchronized with the season). Initial conditions are set at endemic equilibria for model (1) for (**a**), (**c**), (**e**) and $I(0)=0.04$, $R(0)=0.65$ for (**b**), (**d**), (**f**). Notice different scaling on the *y*-axis for figures (**a**), (**b**) and (**c**), (**d**).

**Fig. 9:**
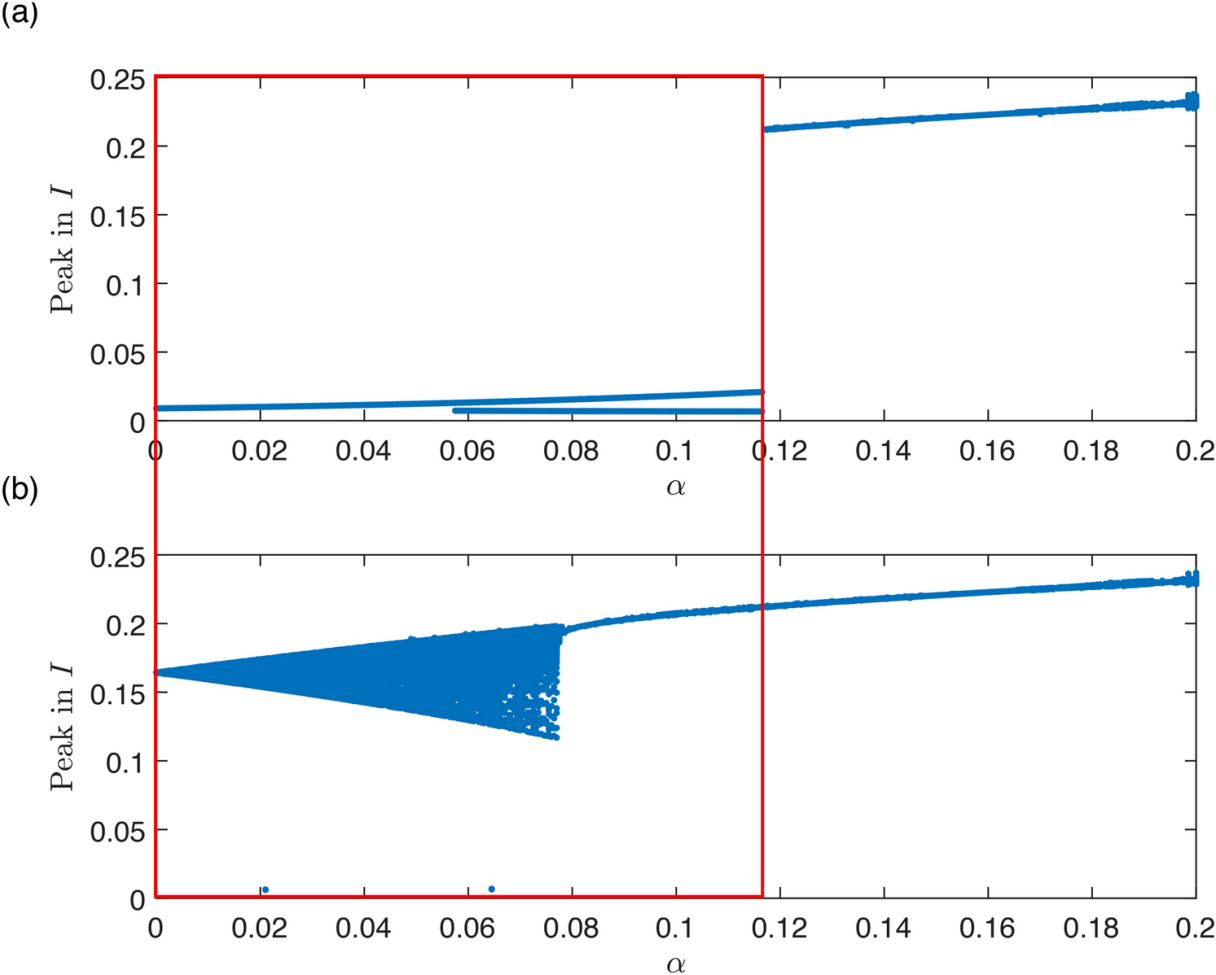
Bifurcation diagram of system (7) with $\alpha$ as parameter and and other parameters are $\beta _0=200$, $K=0.05$, $\gamma =36$, $\eta =2$, $r=100$, $\nu =1$, by excluding transient 100 and with total time 200: (**a**) with initial conditions at endemic equilibria for model (1), (**b**) with initial conditions $I(0)=0.04$, $R(0)=0.65$. Bistability region is marked by red rectangle.

Our approach involves identifying an embedded torus in a multidimensional state space and subsequently locating stable cycles on this torus. This method not only enables us to delineate a specific bistability region within the parameter space (using the parameters $\alpha$ and *K*, though any pair of parameters can be chosen), but also facilitates our understanding of the origin of the bistability region and the estimation of the peak level of the epidemic wave. Additionally, it elucidates the emergence of synchronization with seasonality, including the development of multi-year cycles (that can be found using Neimark-Sacker manifold continuation, which is omitted here for brevity, showing only two regions of period doubling related to 2-year cycles birth). The epidemic cycle is characterized by the state variables *I* and *R*, while the seasonality is described by the state variables *u* and *v* of the system (7). These two coupled cycles form a 2-dimensional torus, with synchronization corresponding to a stable cycle on this torus. This stable cycle emerges in the Arnold tongue as it traverses the limit point of cycle (LPC) manifold. The continuation in the $\alpha$ and *K* parameter space is illustrated in Fig. 6. Here, the sequences of one-parameter continuation sections of the manifold of cycles in *K* and $\alpha$ directions form a typical picture of a twice-folded variety corresponding to the hysteresis phenomenon. The fold corresponds to the LPC, indicating that once the $\alpha$ threshold on this LPC curve is crossed, a transition to seasonally synchronized cycles with a high epidemic peak occurs. This can lead to a significant shift in system dynamics: when initially characterized by minor seasonal fluctuations due to the periodic driving of the endemic equilibrium by the seasonal effect $\alpha$, the system may switch into a highly oscillatory regime and seasonally synchronize. In addition, the hysteresis phenomenon complicates the transition back to a low level of disease prevalence.

**Fig. 10:**
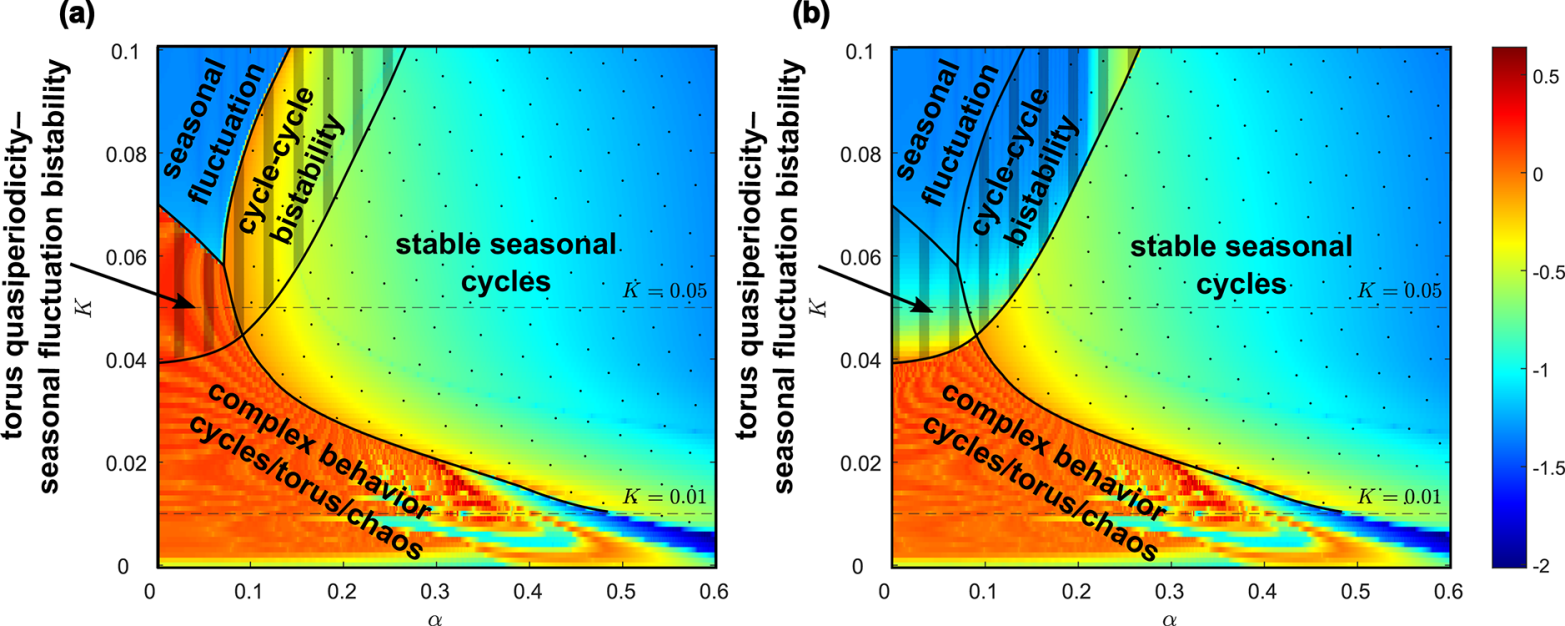
Attractors of system (7) with $\alpha$ and *K* as parameter and and other parameters are $\beta _0=200$, $\gamma =36$, $\eta =2$, $r=100$, $\nu =1$, by excluding transient 50 and with total time 50. Attractors are determined from the Lyapunov spectrum. The initial conditions for state variables *u*, *v* are set so to start on a stable limit cycle. We are excluding the one Lyapunov exponent that is always zero and one that is always negative. We show the maximal Lyapunov exponent of the remaining two (**a**) with initial conditions $I(0)=0.0001$, $R(0)=0.9999$, (**b**) with initial conditions at endemic equilibrium for model (1). Regions characterized by stable seasonally-synchronized cycles are indicated with dots, while regions exhibiting bistability are marked with stripes. By seasonal fluctuation, we mean a limit cycle characterized by small seasonal fluctuations of endemic equilibrium.

Fig. 7 shows two detailed sections. Firstly, for $K=0.01$, Fig. 7(a) illustrates the birth of multi-year cycles and chaos regions, as described in the previous paragraph and Fig. 4. The continuation of the cycle manifold with respect to parameter $\alpha$ reveals the stability of synchronized cycles (determined through multiple computations in MatCont, with stability depicted in blue and instability in red) and bifurcations such as LPC or period-doubling of a cycle (PD). We depict only the maximum of the state variable *I*, which reflects the epidemic peak. The period-doubling route to chaos is represented by the cascade depicted by two branches of 2-year cycles and 4-year cycles below $\alpha \approx 0.4$ (compare with Fig. 4(d)). Notice that the main cycle branch is unstable (red) below this threshold, and the trajectories converge to other attractors, which may be a torus, a chaotic attractor, or another stable cycle born from the LPC or PD point. For more details, see Appendix C and Fig. 10. Secondly, for $K=0.05$, Fig. 7(b) shows the bistability region, as well as the unstable cycle branch (red line) with subcritical period-doubling.

For a better explanation of the behavior in this wide bistability region, we conducted simulations using parameters $\beta _0=200$ (resp. $\beta =200$), $K=0.05$, $\gamma =36$, $\eta =2$, $r=100$, $\nu =1$. The natural frequency of the periodic orbit in model (1) without seasonal transmission rate is approximately 0.94 years. In Figs. 8, 9 you can see that starting with initial conditions sufficiently close to the endemic equilibrium of system (1) the dynamic exhibits small epidemic equilibrium fluctuations with one or two peaks per season, Fig. 8(a) or (c), for small amplitude $\alpha$ of the seasonal effect. Endemic cycles with large seasonal epidemic waves coexisting in the system due to the bistability in system (1) are depicted in Fig. 8(b) or (d). The threshold for bistability is given by subcritical period doubling of the cycle at a seasonality percentage $\alpha \approx 0.16$, but used initial conditions make the system to jump already at a seasonality percentage $\alpha \approx 0.12$ for parameter setting of Figs. 8 and 9. When this threshold is reached the system exhibits exclusively the large seasonal epidemic waves, see Fig. 8(e), (f). For initial conditions that are sufficiently far from the endemic equilibrium of system (1) the system undergoes a transition from a non-synchronized state, see Fig. 8(b), to a synchronized state, see Fig. 8(d), (f), due to LPC threshold at $\alpha \approx 0.08$ for parameter setting of Figs. 8 and 9.

The findings outlined earlier can be concisely presented in Fig. 10. This figure illustrates the maximum Lyapunov exponent, excluding one Lyapunov exponent that consistently equals zero and another that is consistently negative. This exclusion is possible because the initial conditions for *u*, *v* are set to initiate a stable limit cycle, see (7). Fig. 10 illustrates the changes in chaos and quasiperiodicity ranges corresponding to the parameter *K*. No hyper-chaos was detected in the system; at most, one Lyapunov exponent was positive. By comparing Fig. 10(a) and b you can also notice a region of bistability between cycle and torus, and between two cycles, starting from $K\approx 0.04$.

### Unpredictability and stochastic differential equations

**Fig. 11:**
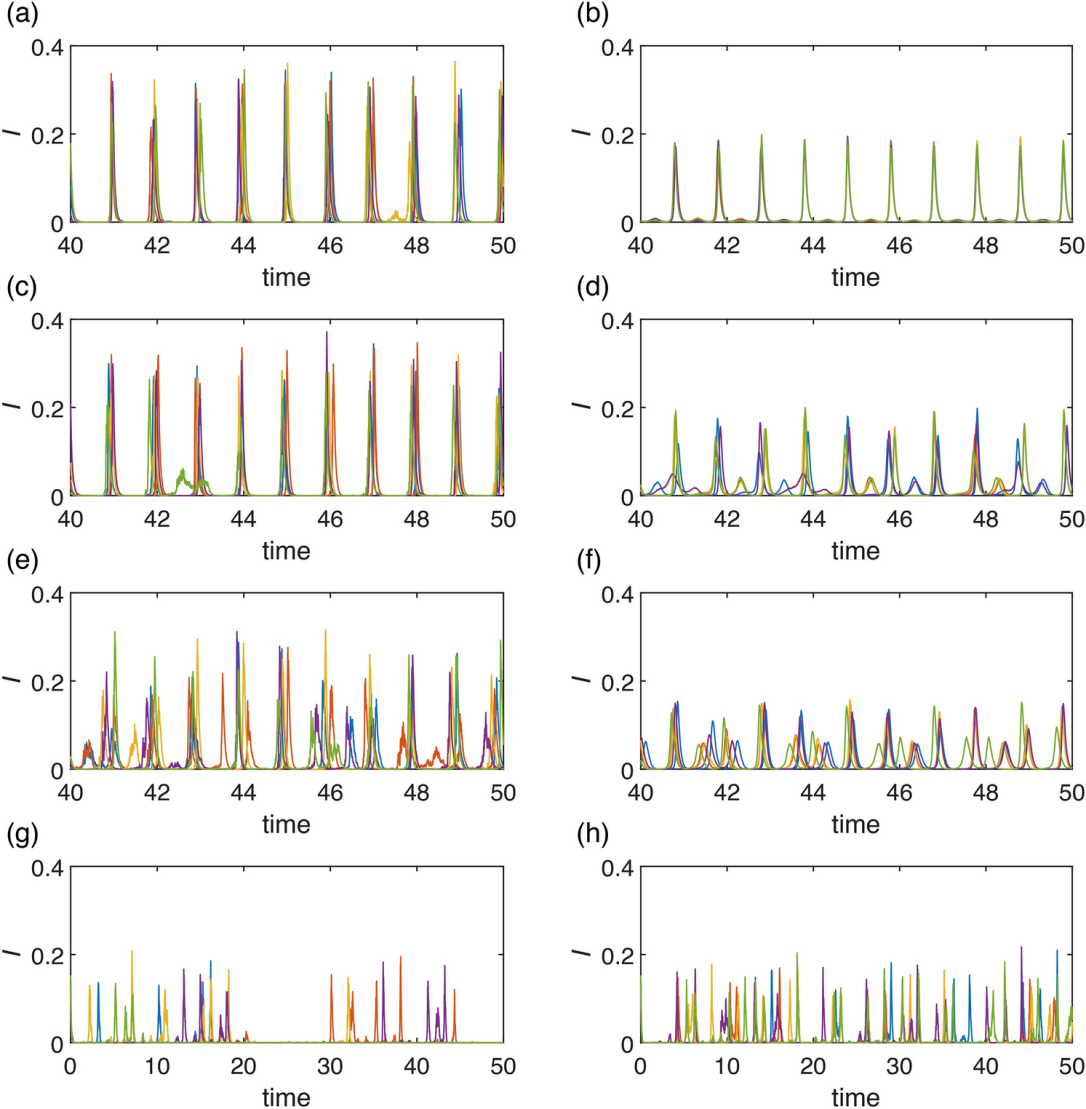
Simulation of stochastic version of model (7) using Milstein method: with parameters $K=0.01$, $\gamma =36$, $\eta =2$, $r=100$, with initial conditions $I(0)=0.15$, $R(0)=0.65$: Seasonal synchronization: (**a**) $\beta =200$, $\alpha =0.45$, $\nu =1$, $\sigma =1$ (**b**) $\beta =200$, $\alpha =0.45$, $\nu =1$, $\sigma =10$; Unpredictability in the region of deterministic chaos: (**c**) $\beta =200$, $\alpha =0.35$, $\nu =1$, $\sigma =1$, (**d**) $\beta =200$, $\alpha =0.35$, $\nu =1$, $\sigma =10$; Unpredictability in the region of quasiperiodicity: (**e**) $\beta =200$, $\alpha =0.2$, $\nu =1$, $\sigma =1$, (**f**) $\beta =200$, $\alpha =0.2$, $\nu =1$, $\sigma =10$; Unpredictability near SNIC and transcritical bifurcations: (**g**) $\beta =60$, $\alpha =0.45$, $\nu =4$, $\sigma =2.5$, (**h**) $\beta =60$, $\alpha =0.45$, $\nu =4$, $\sigma =2$. Notice different scaling on *x*-axis for figures (**a)**–(**e**) and (**g)**, (**h**).

In this section, we introduce the consideration of stochasticity in the transmission rate, acknowledging that its fluctuations are not entirely deterministic. We present mechanisms of how stochastic deviations from deterministic dynamics can lead to dynamical unpredictability. They are based on previous rigorous bifurcation analysis that revealed various possibilities of abrupt changes and various bistability regions. In case of structurally stable deterministic dynamics, small stochastic change has no significant effect to dynamics and does not influence predictability. But near bifurcation thresholds, separatrices of basins of attraction in bistability regions, or in chaotic region, the dynamics predictability is lost or at least depreciated. Moreover, quasiperiodicity on torus may look unpredictable in a short period even in a deterministic case. To show these mechanisms, we simulate model (8).

When simulating the dynamics in areas characterized by strong seasonal synchronization, we note only minimal variability in peak height and occasional emergence of a secondary outbreak (spring or summer), see Fig. 11(a). In such scenarios, a lower variability $\sigma$ in transmission rate contributes to stabilizing the dynamics. The secondary outbreak may then occur every year, refer to Fig. 11(b).

**Fig. 12:**
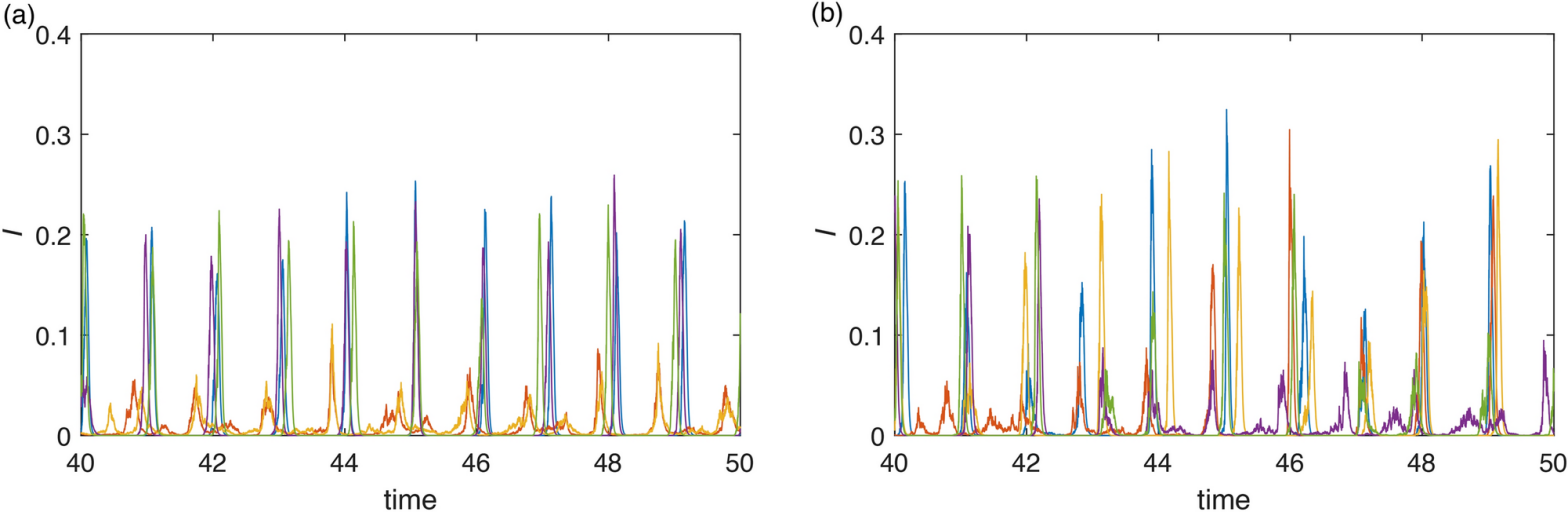
Simulation of stochastic version of model (7) using Milstein method: with parameters $K=0.1$, $\gamma =36$, $\eta =2$, $r=100$, $\beta =200$, $\alpha =0.45$, $\nu =1$ with initial conditions $I(0)=0.15$, $R(0)=0.65$: Seasonal synchronization: (**a**) $\sigma =5$, (**b**) $\sigma =8$.

Our primary focus lies in regions where the dynamics of our model demonstrate high complexity. Here, we identify four distinct sources of unpredictability:

1. *Unpredictability in the region of deterministic chaos*
  The first source is deterministic chaos, a well-known phenomenon leading to unpredictable dynamics^38,44,45^. However, within the context of seasonal synchronization, we observe that although the solution displays chaotic behavior-meaning the number and magnitude of peaks are unpredictable-the connection to the year-long season is not lost for strong enough seasonality, see Fig. 11(d). Additionally, we note that higher variability $\sigma$ in the transmission rate can have a stabilizing effect on seasonal synchronization, see Fig. 11(c). Although predicting the amplitudes of the winter outbreaks is not possible, their occurrence remains predictable as an epidemic phenomenon (due to chaotic attractor existence and shape).
2. *Unpredictability in the region of quasiperiodicity*
  The second source is quasiperiodicity, which arises when seasonal synchronization is not sufficiently strong, meaning for small $\alpha$, see Fig. 5. In such cases, the natural frequency of model (1) prevents epidemic dynamics from synchronizing with the seasons, resulting in random outbreaks throughout the year, see Fig. 11(e), (f). In this case, fluctuations in the transmission rate result in the unpredictability of both the amplitudes and the time onsets of the outbreaks.
3. *Unpredictability near SNIC and transcritical bifurcations*
  The third source is the closeness of a bifurcation point. In our proposed model, two bifurcations play crucial roles in the unpredictability of the epidemic dynamics. The first is the SNIC bifurcation, wherein the period of the limit cycle rapidly increases in its vicinity, leading to unpredictability in peak locations. The second bifurcation is transcritical, which may result in the eradication of the disease. Both those phenomena can be observed in Fig. 11(g), (h). For larger variability $\sigma$, see Fig. 11(g), the suppression of the epidemic is more likely. The possibility of crossing the threshold, as given by SNIC or transcritical bifurcation, introduces high unpredictability in the timing of outbreak onset or the eradication of the disease.
4. *Unpredictability due to bistability*
  Another possibility is abrupt crossing of basins of attraction in bistability region. This is also a case of a deterministic system. Fig. 12(a), (b) depicts stochastic occurence of low or high levels of epidemic peaks with possible unpredictable transients (b).

### Empirical validation: climate-specific influenza dynamics and post-pandemic seasonality

In this subsection, we applied our approach to model the influenza epidemic characteristics^13^. Data from various countries can be categorized into three climate groups: Temperate, Subtropical, and Tropical. From the perspective of our model, these groups are characterized by different levels of seasonality $\alpha$. Specifically, the temperate climate exhibits high seasonality, the subtropical climate shows moderate seasonality, and the tropical climate has the lowest level of seasonality.

**Fig. 13:**
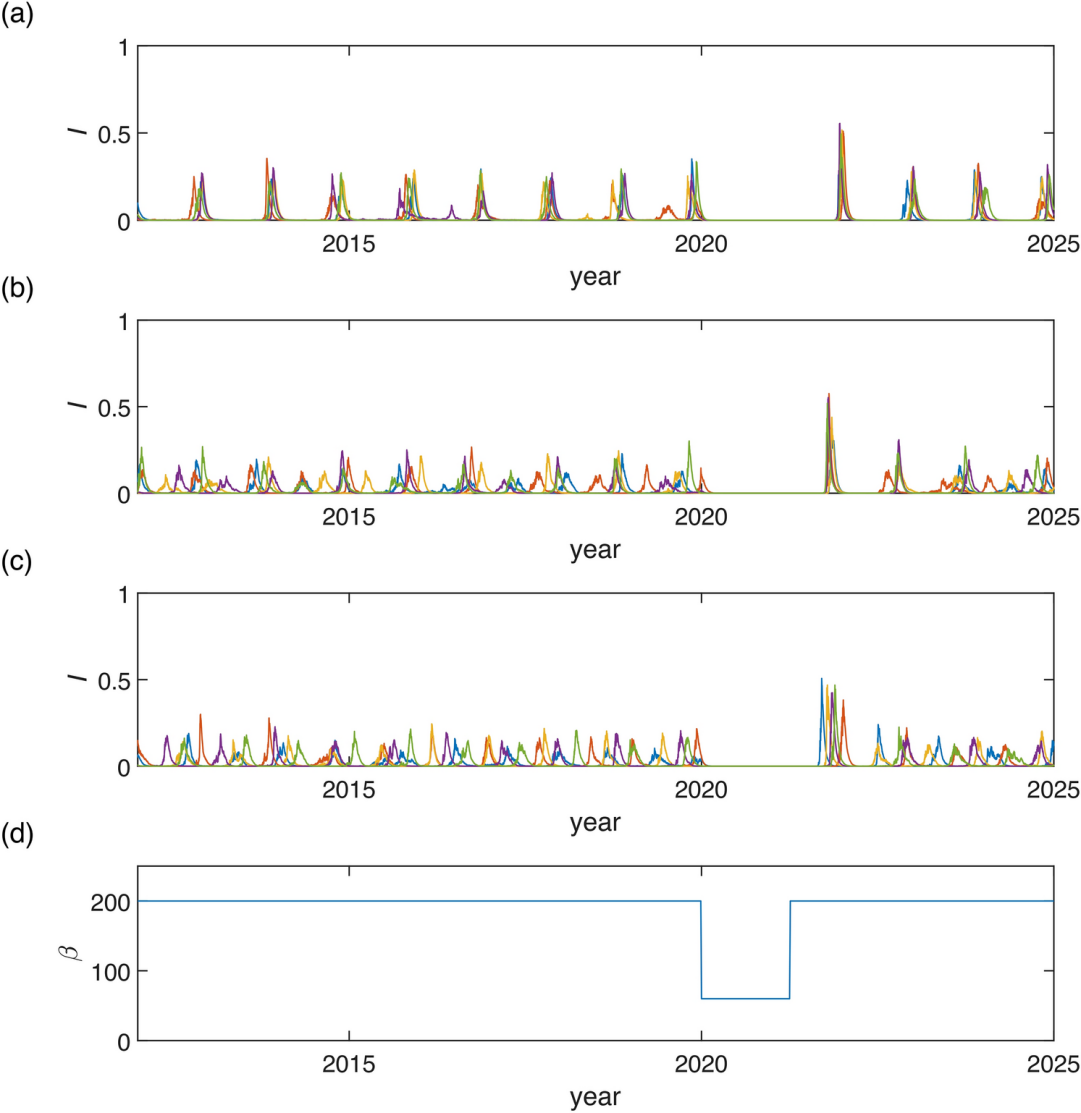
Simulation of stochastic version of model (7) using Milstein method: with parameters $K=0.1$, $\gamma =36$, $\eta =2$, $r=100$, $\nu =1$, $\sigma =10$, using two different levels of parameter $\beta$: $\beta =200$ and $\beta =60$ during the simulation (see (**d**)), with initial conditions $I(0)=0.15$, $R(0)=0.65$. (**a)**–(**c**) shows different levels of seasonality (**a**) Temperate climate: $\alpha =0.3$, (**b**) Subtropical climate: $\alpha =0.15$, (**c**) Tropical climate: $\alpha =0.05$.

The simulation results, see Fig. 13, obtained using the Milstein method, where Non-pharmaceutical interventions (NPIs) were modeled by decreased transmission rate $\beta$ during the first two years of COVID-19 pandemics to replicate real influenza positive rates by climate zones (compare with real data presented in Fig. 1 of the original study^13^ or with WHO Influenza Surveillance Reports^46^). Notably, after the release of NPIs, the first peak in disease incidence was higher than the peaks observed before the pandemic, but it subsequently decreased, aligning with observations^46^.

Our simulation accurately captures the dynamics observed across different climate groups. Specifically, for countries with a Temperate climate, the disease’s strong seasonal pattern, characterized by high synchronization, was reestablished after the relaxation of NPIs. In contrast, for Subtropical and Tropical climates, the seasonality was not always strong enough to consistently produce synchronization, with some countries exhibiting synchronization while others showed it less prominently or not at all, both before and after the pandemic. This pattern is consistent with our simulation results, demonstrating that while Temperate regions quickly regained seasonal synchronization, the other climate groups maintained a more variable pattern of synchronization throughout. Although the model parameters were chosen to resemble the dynamics of COVID-19, other respiratory diseases exhibit similar dynamics. Given the phenomenological nature of our model, it is sufficient for capturing the observed dynamics.

## Discussion

In our paper, we aimed to study the synchronization between epidemic outbreaks and seasons. Hence, we selected a model that undergoes a transition towards oscillations as our starting point to examine its interactions with seasonal cycles. We also aimed to choose a model that is applicable to the currently studied epidemic disease, with parameters that can be (at least roughly) set. Consequently, it is reasonable to begin with a model that accounts for a threshold in healthcare capacity^2^. When we set the parameters of the model, we obtained non-seasonal epidemic cycles that are close to one year in length. We modeled seasonality with simple harmonic oscillations because it is the easiest possible way, consistent with approaches used by other authors in the past^7^.

Although the epidemic model employed in this study and the implementation of seasonal forcing follow standard formulations, our focus lies in the analysis of the resulting nonlinear dynamics beyond the commonly studied modulation of endemic equilibrium fluctuations^7^. Specifically, we examine how the intrinsic epidemic cycle, already oscillatory in the autonomous case, interacts with seasonal forcing. This interaction can lead to a range of dynamical regimes, including phase locking, quasiperiodicity, and desynchronization, which we characterize using Lyapunov exponents. Drawing from previous work in neuroscience^47–50^, physics^35,51,52^, and population dynamics^36,53,54^, we believe that these findings are largely model-independent and represent a generic scenario in seasonally driven nonlinear systems.

It is important to note that our approach does not consist of simulations but rather bifurcation analysis, although computed numerically. Numerical algorithms thus continue uniquely defined cycle manifolds in the parameter space, guaranteed by the theorem on continuous dependence on parameters and initial conditions, as well as by the genericity of bifurcations. The accuracy of these computations can be set arbitrarily within the employed numerical methods. The key advantage of this approach is that it does not rely on the precise selection of parameters, as qualitatively the same dynamic behavior can be observed across a wide range of parameter values. Due to known characteristics of COVID-19 epidemics^25–28^, we chose parameters within a range that corresponds to an actual real Acute Respiratory Infection (ARI) disease. We conducted a two-parameter analysis to systematically identify the boundaries where changes in dynamics occur, demonstrating that small variations in parameters within generic regions do not disrupt the continuity of dynamic structures, while significant transitions occur only at bifurcation points of higher codimension, which we detect and describe in our analysis. While our model is framed within the context of COVID-19, the results are generic and applicable to a wide range of respiratory diseases.

In our model (1), we replaced the original step function^2^ with a smoother sigmoidal function *f* (Eq. 2) to better capture the prevalence threshold that triggers changes in infectiousness duration. This modification preserves the original model’s dynamics for steep slopes (as controlled by parameter *r*) while allowing greater flexibility for scenarios like healthcare capacity, asymptomatic cases, and non-pharmaceutical interventions. The smoothness of *f* not only reflects gradual changes in infectiousness more realistically, but also stabilizes numerical solvers and enables bifurcation analysis, which would not be possible with a non-smooth function.

Our model (7) shows chaotic dynamics across a broad spectrum of parameters. However, strong seasonality holds the potential to stabilize this chaos on an attractor that despite its chaotic character stays synchronized with the season. This implies an interesting phenomenon within the chaotic regime, where unpredictability predominantly emerges in the maximum peaks of outbreak occurrences. The system maintains a close association with the seasonal cycle, with a significant likelihood of major outbreaks during the winter season.

Additionally, model (7) also exhibits quasiperiodicity. This refers to dynamics on an invariant torus within parameter space, where epidemic outbreaks and seasons lack synchronization. Consequently, an epidemic outbreak may occur at any point throughout the year. In terms of outbreak timing, this could be even more unpredictable than deterministic chaos, owing to external random effects on the epidemics. Moreover, in case of a new unknown disease, the outbreaks may seem to be random as the reported timeseries are too short. As you can see in Fig. 5(b), it is hard to distinguish between chaos and quasiperiodicity. On the other hand, as discussed in “Unpredictability and stochastic differential equations” section, both chaos and quasiperiodicity are sources of unpredictability.

Moreover, saddle-node infinite cycle (SNIC) and transcritical bifurcations seem to become another source of unpredictability as the system dynamics is getting close to eradication, as discussed in “Unpredictability and stochastic differential equations” section. Approaching the SNIC bifurcation due to vaccination (resulting in a decrease in transmissibility rate) presents a scenario of gradual eradication that aligns qualitatively with pertussis data from the United States or Europe following the implementation of mandatory vaccination against pertussis in the mid-20th century^55,56^. Moreover, various inter-epidemic periods of pertussis are found during the early vaccine era in the US^14^, and three dynamical categories of outbreaks in different states are set: with annual, initially annual and later multiannual, or multiannual outbreaks. This is absolutely in accordance with our model with an endemic cycle, as the SNIC bifurcation critical point is reached and crossed in each state at different times with respect to the percentage and time schedule of vaccine uptake. The same mechanism explains the prolongation of the inter-epidemic period of pertussis with respect to vaccine-uptake all over the world^15^.

The last dynamical phenomenon we discuss in the paper is bistability. Bistability poses significant risks due to the potential for abrupt transitions between endemic cycles triggered by local superspreading events or migration. Our analysis reveals that, within the larger endemic cycle, the incidence and prevalence of a disease could escalate by an order of magnitude. The scales depicted in Fig. 8(a)–(d) illustrate almost doubled percentage of individuals affected by the disease over a one-year span, and more than a tenfold increase in the disease prevalence during the epidemic peak. This phenomenon may represent another mechanism or contribute additively to the emergence and stabilization of large outbreaks. This is in addition to the variability in individual infectiousness^57^ and numerous biological, socio-behavioral, and environmental factors that influence the basic reproduction number^58^, potentially causing significant initial fluctuations in prevalence.

We performed an empirical validation of our results by applying the proposed model to a real-world problem: modeling the characteristics of influenza epidemics across different climate zones. The simulation results, which accurately reflect observed epidemiological patterns, demonstrate the model’s ability to capture the distinct dynamics of disease spread in Temperate, Subtropical, and Tropical climates across pre-pandemic, pandemic, and post-pandemic periods, including the effects of non-pharmaceutical interventions (NPIs)

While our model provides valuable insights into epidemic dynamics, it also comes with certain limitations that warrant acknowledgment. Firstly, we simplify the model by omitting the exposed compartment (E), which is related to the latency period, potentially oversimplifying the real-world scenario. This omission may cause small shifts in peak timing, which is observed in reality, but has no significant impact on studied phenomena, since it causes a delay only. Additionally, we do not distinguish between recovered (R) and quarantined compartments (Q), which could affect the accuracy. The principles of our analysis would remain the same even if compartment Q was incorporated. Moreover, the model does not account for deaths resulting from the disease, which may be a crucial aspect in understanding its impact. On the other hand, it is worth noting that the vital dynamics, while discussed, have only a minor effect on the model outcomes.

Although our model may have its limitations, the application of bifurcation theory in epidemiology presents a significant opportunity for gaining insights into the intricate dynamics of infectious diseases. Further exploration could involve investigating alternative models exhibiting endemic cycles, exploring the potential of utilizing bifurcation theory for optimizing vaccine uptake scheduling, particularly among children populations, and aligning with the school year cycle. Another promising research direction involves modeling cross-interactions related to the spread of certain diseases between human populations in different geographical units (such as states, cities, districts, and regions), an approach commonly used in population dynamics to capture spatial interactions and migration patterns^59,60^. Additionally, there is potential in Partially Observed Markov Processes (POMP) techniques to effectively fit real-world data, paving the way for more accurate epidemiological predictions and interventions^61,62^.

In conclusion, we have tried to mimic the complex dynamics of real diseases in the strategic choice of model parameters. However, upon rigorous analysis, we encountered a parameter region where predictability decreases and admits various sources of unpredictability, which could be related to a possible evolutionary advantage of such a parasite-host arrangement. Whether this finding goes beyond mere chance and suggests a fundamental principle in terms of eco-epidemiology remains an open question.

## Figures and Tables

**Fig. 14:**
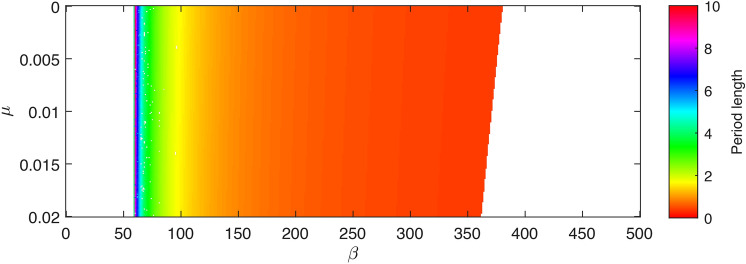
Period lengths of limit cycle solution of system (9) with $\beta$ and $\mu$ as parameters, $\lambda =1.015\,\mu$, and other parameters are $K=0.01$ $\gamma =36$, $\eta =2$, $r=100$, $\nu =1$. Bifurcation diagram and period for $\mu =0$ is in Fig. 2(a), (d). Red color indicates very short period close to zero (zero cannot be reached).

**Fig. 15:**
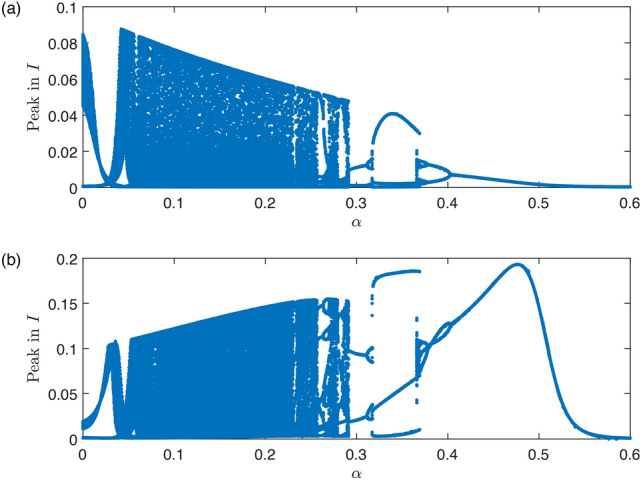
Bifurcation diagram of system (7) with $\alpha$ as parameter and and other parameters are $K=0.01$, $\beta =200$, $\gamma =36$, $\eta =2$, $r=100$, $\nu =1$, with initial conditions $I(0)=0.15$, $R(0)=0.65$, transient 100 and total 200: (**a**) section of the epidemic cycle at $t=0.3+n$, where integer $n \in \left\{ 100, \dots , 199\right\}$, (**b**) section of the epidemic cycle at $t=0.8+n$, where integer $n \in \left\{ 100, \dots , 199\right\}$.

**Fig. 16:**
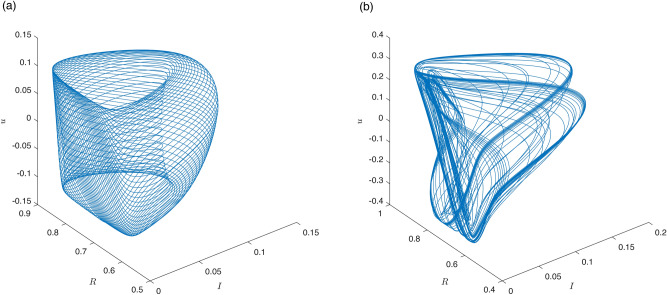
Two seasonally non-synchronized attractors of the system (7) with parameters $\beta =200$, $K=0.01$, $\gamma =36$, $\eta =2$, $r=100$, $\nu =1$: (**a**) torus with a quasiperiodic trajectory for $\alpha =0.1$, (**b**) chaotic attractor for $\alpha =\sqrt{0.1}$.

## Data Availability

The datasets generated during and/or analysed during the current study are available in the Gitlab repository: https://gitlab.ics.muni.cz/ndteam/epidemiology/synchronization-with-season

## Appendix

### A: Limited treatment model with vital dynamics

To enhance the realism of the limited treatment model (1), we suggest incorporating vital dynamics. In this proposed scenario, infected parents give birth to infected offspring, while both recovered and susceptible individuals give birth to offspring susceptible to the infection. All individuals in these three compartments experience the same rates of mortality ($\mu$) and birth ($\lambda$). As a result, the following equations emerge:

9 $$\begin{aligned} \begin{aligned} \frac{dS}{dt}&= -\beta SI + \nu R + \lambda (S + R) - \mu S ,\\ \frac{dI}{dt}&= \beta SI - \gamma f(K,\eta ,r,I)I + (\lambda - \mu )I, \\ \frac{dR}{dt}&= \gamma f(K,\eta ,r,I)I - \nu R - \mu R. \end{aligned} \end{aligned}$$

For human population both the mortality rate $\mu$ and birth rate $\lambda$ are relatively small. Let us assume $\mu \in [0, 0.02]$, and $\lambda =1.015 \,\mu$ in accordance with^63,64^.

As outlined in the main text, the period length of the periodic solution of model (1) is crucial for synchronizing with the seasons. Consequently, our focus is on evaluating the impact of vital dynamics on the period length in model (9). Given that both the mortality rate $\mu$ and birth rate $\lambda$ are relatively small, resulting in only minor changes in the period length as depicted in Fig. 14, we choose to disregard this factor.

### B: Sections of the epidemic cycle

Identifying the local peaks of a limit cycle concerning the state variable *I* might not be as accurate as segmenting the solutions. Furthermore, the limit cycle could display multiple peaks even before undergoing period-doubling bifurcation, as depicted in Fig. 4(e), (f). Hence, we offer segments of the epidemic cycle at two different times of the year, as illustrated in Fig. 15. However, accurately identifying these peaks is directly relevant to epidemiology.

### C: Torus and strange chaotic attractor

For low levels of *K* and $\alpha$, the endemic cycle of the system (7) cannot synchronize with the season. There are two possible attractors that appear in the system (7) aside from stable cycles. One is the embedded invariant torus that appears when the coupling $\alpha$ is too small with respect to *K* (for values of $\alpha$ lower than the threshold on the LPC curve), and the second is a chaotic attractor. Their emergence in the $\alpha \times K$ plane is described in Fig. 10. Fig. 16 shows these typical attractors by displaying trajectories that reside on them for parameter $\alpha =0.1$ (torus) and $\alpha =\sqrt{0.1}$ (strange chaotic attractor).
